# Diurnal and semidiurnal internal waves on the southern slope of the Yermak Plateau

**DOI:** 10.1038/s41598-022-15662-0

**Published:** 2022-07-08

**Authors:** Shuya Wang, Anzhou Cao, Qun Li, Xu Chen

**Affiliations:** 1 Key Laboratory of Physical Oceanography, Ocean University of China and Pilot National Laboratory for Marine Science and Technology, Qingdao, China; 2 MNR Key Laboratory for Polar Science, Polar Research Institute of China, Shanghai, China; 3 Ocean College, Zhejiang University, Zhoushan, China

**Keywords:** Physical oceanography, Physical oceanography

## Abstract

The Yermak Plateau (YP) is located across the Arctic–Atlantic gateway in the northwest of the Svalbard archipelago. In this region, internal waves are believed to cause intense turbulent mixing and hence influence the heat budget in the Arctic Ocean. Based on year-long observations from three moorings, the characteristics and energetics of diurnal and semidiurnal internal waves on the southern slope of the YP are investigated in this study. Diurnal internal waves induce large isothermal displacements exceeding 100 m, which are nearly one order of magnitude greater than those of semidiurnal internal waves. In addition, diurnal internal waves are strong in winter but weak in summer, while the semidiurnal internal waves exhibit complicated temporal variation. For the diurnal internal waves, their available potential energy is greater than the horizontal kinetic energy; whereas the situation is opposite for the semidiurnal ones. This feature is further clarified with two-dimensional numerical simulations. Due to the larger tidal excursion, diurnal tidal forcing yields the generation of stronger higher harmonics, i.e., the semidiurnal internal waves. In contrast, higher harmonics are rather weak under the semidiurnal forcing. Moreover, a large proportion of energy for both diurnal and semidiurnal internal waves is dissipated locally. Results of this study can provide useful insight on the dynamics of internal waves in the Arctic Ocean.

## Introduction

As an important intermediate step of the oceanic energy cascade, internal waves make a significant contribution to the turbulent mixing, which is essential for the maintenance of global overturning circulation^1–4^. In the mid-latitude ocean, tide-generated internal tides and wind-generated near-inertial internal waves make comparable contributions to the mixing^5–10^. However, in the Arctic Ocean (AO), because of the presence of sea ice, the wind energy importing into the ocean is damped to an extent^11–14^, which, on the other hand, highlights the role of tides in driving the mixing^15^.

Previous observations have demonstrated wide-spread internal tidal waves within diurnal and semidiurnal bands in the AO^16–22^. Because most of the AO is poleward of the critical latitudes (where the local inertial frequency is equal to the tidal frequency) of both semidiurnal and diurnal tides, internal tidal waves cannot freely propagate far away from their generation sites. Instead, they are trapped near their generation sites under the constraint of rotation and dissipated locally^23–26^, resulting in enhanced mixing in some slope regions in the AO^20,21,26–28^. The enhanced mixing further affects the heat budget in the AO and influences the global climate^15,29,30^.

**Figure 1:**
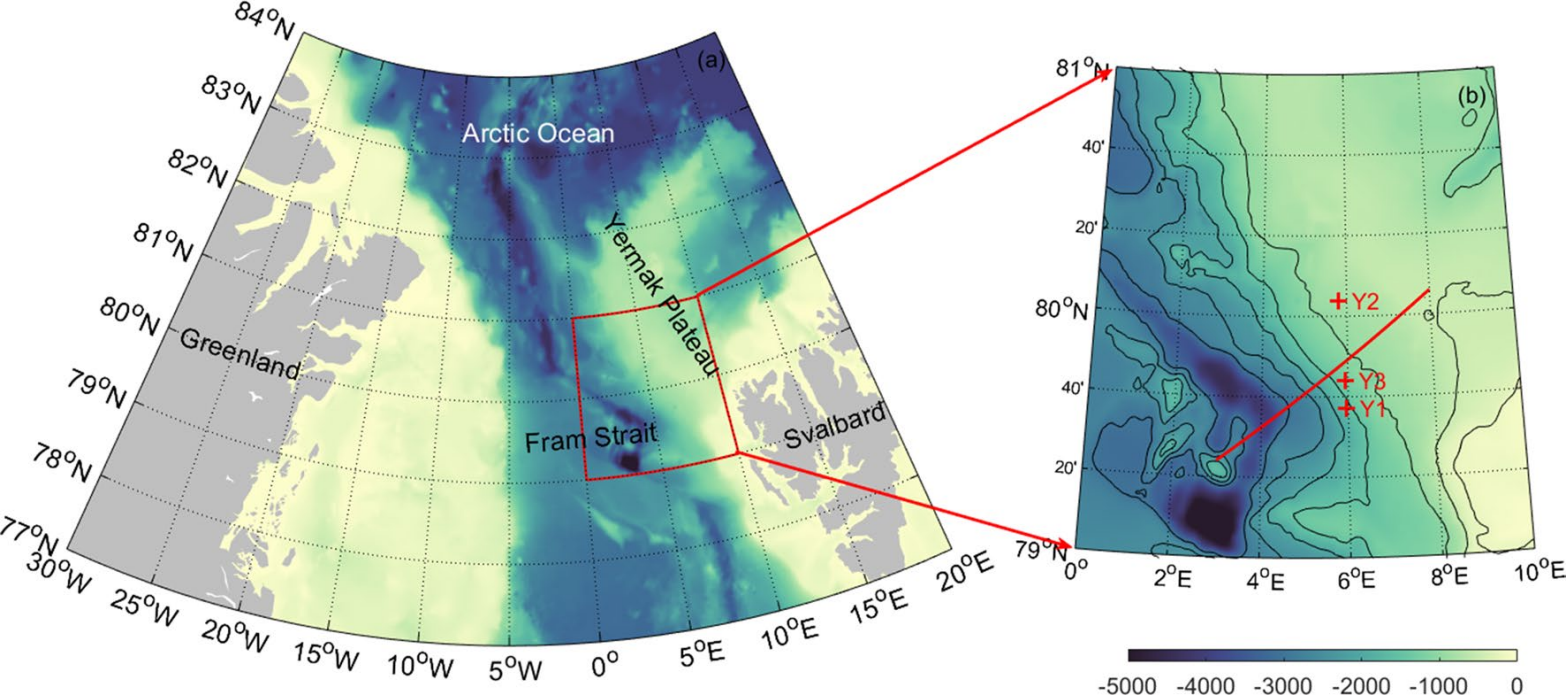
(**a**) Location map of the study region with the southern slope of YP marked by the red box. Bathymetry from ETOPO1 is indicated by shading (unit: m). (**b**) Enlarged map of the southern slope of YP. Isobaths are denoted by black contours. Mooring locations are indicated by red pluses. The red line indicates the topographic transect used in the numerical model.

The Yermak Plateau (YP) is the main passage of the Atlantic Water to the AO (Fig. 1a;^28,31^). The Atlantic Water carried by the West Spitsbergen Current could be an important source of heat and salt for the AO^32^. As the Atlantic Water flows over the YP, it gets cooled and freshened because of strong air-sea fluxes and turbulent mixing^15,21,28^. Given the presence of strong barotropic tidal flow and steep slopes around the YP^33^, breaking of tide-induced internal waves is considered to play a dominant role in driving the mixing near the YP^21^.

Early observations from Padman et al.^16^ have verified the presence of internal tidal waves at the YP, which could induce intense current and isothermal displacement. The following work^20^ indicates that the internal tidal waves in this region are mainly in the diurnal and semidiurnal bands. Also, the measured dissipation rates at the YP are much higher than those in deep basin of AO^27,28,34^. The simulated barotropic-to-baroclinic energy conversion in this region reaches 1 GW^21^, which roughly accounts for 20% of the total conversion in the AO (~ 5 GW^7,10^). It is also found that the internal tidal waves around the YP behave as trapped waves, and a large proportion of their energy is dissipated locally, which is responsible for the observed enhanced mixing^21^. These studies substantially deepen our understanding of internal waves in the AO. However, owing to limited observations (site, range and duration), there remain some unknowns about the internal waves in the AO.

In this work, the diurnal and semidiurnal internal waves on the southern slope of the Yermak Plateau are investigated. Field experiment conducted in 2014–2015 provides valuable data on the southern slope of the YP^35,36^, which give us an opportunity to better understand the internal waves there. During the experiment, year-long data of currents, temperature and salinity were collected at three moorings with high temporal resolution and large vertical range. Based on these observations, the characteristics and energetics of diurnal and semidiurnal internal waves on the southern slope of the YP are analyzed. Moreover, two-dimensional numerical simulations are conducted, aiming to further investigate internal wave dynamics in this region.

## Method

### Moored observations

Observations from three moorings (Y1–Y3^36^) during September 2014–August 2015 were used to study the internal waves on the southern slope of the YP. Figure 1b shows the locations of the three moorings, and more detailed information about the moorings is listed in Table S1 in supplementary information. At each mooring, the oceanic currents, temperature and salinity were recorded, with raw observations shown in Figs. S1–S3. All the observed data were interpolated at uniform 5 m vertical levels and one-hour temporal intervals.

Because the moored observations cannot cover the full water depth at the three moorings, the temperature and salinity data from Multi Observation Global Ocean 3D Temperature Salinity Height Geostrophic Current and MLD products (MULTIOBS) at the three moorings are used as a supplementary. Results indicated that the MULTIOBS temperature, salinity and density show good agreements with observations (Fig. S4). In addition, barotropic tidal currents from Arc5km2018^37^ are also used as a comparison with observations.

### Data processing

Gaps exist in the raw observations, which may be caused by the vertical excursion of instruments under the influence of water movements. Here, linear interpolation is adopted to fill in the gaps of current observations in the temporal domain, if the raw current observations covered more than 95% of the total observation period at the corresponding depth^38^. After this processing, the effective ranges of currents at moorings Y1-Y3 are 85–1095 m, 50–705 m and 75–1035 m, respectively.

To isolate diurnal and semidiurnal signals from raw observations, a forth-order Butterworth filter is employed. The cutoff frequencies for diurnal and semidiurnal currents are [0.80, 1.20] cpd and [1.73, 2.13] cpd, respectively, which are determined from power spectra of currents. Note that wind-generated near-inertial waves are included in semidiurnal signals. Removing them from semidiurnal signals could be difficult, since their frequencies are close to the M_2_ and S_2_ tidal frequencies and considerably modulated by background currents^39^.

The vertical displacements at the moorings are estimated from temperature rather than density, because of the sparse observations of salinity. Here, isotherm displacement is calculated by $\eta (z,t) = [T(z,t) - \overline{T} (z,t)]/T_{z} (z,t)$, where $\overline{T} (z,t)$ is the background temperature and $T_{z} (z,t)$ is the temperature gradient^40,41^. Both $\overline{T} (z,t)$ and $T_{z} (z,t)$ are calculated with low-passed temperature considering the slow-varying background stratification^42^.

The filtered diurnal and semidiurnal signals are projected onto vertical modes. As indicated in recent studies, this method is also feasible poleward of the critical latitude^21,23,25,26^. From the modal prospective, horizontal currents (*u* and *v*, corresponding to the zonal and meridional components, respectively) and vertical displacements (*η*) of internal waves can be expressed as:

1 $$\left\{ {\begin{array}{*{20}l} {u^{\prime}\left( {z,t} \right) = \sum\limits_{n = 0}^{{N_{m} }} {u_{n} \left( t \right)\Pi_{n} \left( z \right)} } \hfill \\ {v^{\prime}\left( {z,t} \right) = \sum\limits_{n = 0}^{{N_{m} }} {v_{n} \left( t \right)\Pi_{n} \left( z \right)} } \hfill \\ {\eta \left( {z,t} \right) = \sum\limits_{n = 1}^{{N_{m} }} {\eta_{n} \left( t \right)\Phi_{n} \left( z \right)} } \hfill \\ \end{array} } \right.,$$

where Φ_n_ are the eigenfunctions of the eigenvalue problem for eigenspeed cn:

2 $$\frac{{{\text{d}}^{2} \Phi_{n} }}{{{\text{d}}z^{2} }} + \frac{{N^{2} }}{{c_{n}^{2} }}\Phi_{n} = 0 ,$$

subject to boundary conditions Φ_n_(0) = Φ_n_(− H) = 0,

3 $$\Pi_{n} \left( z \right) = \rho_{0} c_{n}^{2} \frac{{{\text{d}}\Phi_{n} \left( z \right)}}{{{\text{d}}z}} ,$$

*z* is the depth, *t* is the time, *N* is the buoyancy frequency, *ρ*_0_ = 1027 kg/m^3^ is the reference density, *H* is the local water depth, *u*_n_, *v*_n_ and *η*_n_ are the amplitudes of modal horizontal currents and vertical displacements with respect to mode *n* (*n* = 0 for the barotropic mode and *n* > 0 baroclinic modes). Following Alford and Zhao^40^, *N*_m_ = 2 was considered in this study. Moreover, due to seasonal variation of background stratification, monthly averaged *N* from MULTIOBS is used in modal decomposition. A validation of modal decomposition is performed in the supplementary information (Text S1).

After modal decomposition, full-depth horizontal currents and vertical displacements at the moorings are obtained, which can be used to calculate the depth-integrated horizontal kinetic energy (HKE) and available potential energy (APE) for internal waves:

4 $${\text{HKE}}\left( t \right) = \frac{1}{2}\rho_{0} \int_{ - H}^{0} {[u^{\prime}(z,t)^{2} + v^{\prime}(z,t)^{2} ]{\text{d}}z},$$

and

5 $${\text{APE}}\left( t \right) = \frac{1}{2}\rho_{0} \int_{ - H}^{0} {N\left( {z,t} \right)^{2} \eta \left( {z,t} \right)^{{2}} {\text{d}}z} ,$$

### Numerical modelling

The Massachusetts Institute of Technology general circulation model (MITgcm^43^) is used in this study, which solves fully nonlinear governing equations under the Boussinesq approximation with finite-volume method. The propose of our simulation is to clarify the internal wave dynamics captured by three moorings, rather than to reproduce the observations. A two-dimensional configuration of the model is considered, since the cross-isobath tidal current is dominant for barotropic tides. Along-isobath velocity is also allowed in the model due to the presence of Coriolis terms in horizontal momentum equations. In addition, the model is run in a hydrostatic mode, because the highly nonlinear internal waves as in Vlasenko et al.^44^ and Rippeth et al.^25^ are beyond the scope of this study. Horizontal resolution is set to 100 m near the slope region, and it is linearly stretched to 6 × 10^3^ m at open boundaries. Vertical resolution is set to 10 m. The total grid number of the simulation is 2000 × 350. Flux-limited scheme is applied to the advection of temperature and salinity. Bottom boundary is treated as free-slip since boundary layer process cannot be resolved with the present vertical resolution^45^. Sponge layers are added at two open boundaries to avoid boundary reflection of baroclinic waves. In addition, other parameters in the model are listed in Table 1, which are chosen empirically to suppress grid-scale instability^46–48^.

**Table 1:** Parameters in the MITgcm.

Name	Notation	Value
Horizontal resolution	∆*x*	10^2^ ~ 6 × 10^3^ m
Vertical resolution	∆*z*	10 m
Domain length	*L*	640 km
Domain depth	*H*	3500 m,
Horizontal viscosity	*A*_h_	1 m^2^/s
Vertical viscosity	*A*_v_	10^–3^ m^2^/s
Horizontal diffusivity	*K*_h_	10^–3^ m^2^/s
Vertical diffusivity	*K*_v_	10^–5^ m^2^/s
Coriolis frequency	*f*_0_	1.43 × 10^–4^ s^-1^
Time step	∆*t*	2.5 s

Realistic topography is extracted from ETOPO1 along the transect in Fig. 1b. The maximal and minimal depth are set to 3500 and 500 m, respectively. The maximal slope of the topography is 0.09. Stratification is derived from the MULTIOBS. Tidal forcing is imposed by adding a body force to the horizontal momentum equations, as in previous studies^25,49–51^. Single tidal forcing is considered, whose amplitude *U*_0_ is obtained from observed barotropic currents at Y1 by harmonic analysis. Each simulation is operated for 6 days, and the simulated results are output every half an hour. Several sensitivity runs with different tidal forcing and stratifications are designed, and detailed information is shown in Table 2.

**Table 2:** Parameters in the sensitivity experiments.

Name	Forcing frequency (s^-1^)	Amplitude (m/s)	Stratification
K1A	7.29 × 10^–5^	0.031	Annual
K1W	7.29 × 10^–5^	0.031	Winter
K1S	7.29 × 10^–5^	0.031	Summer
M2A	1.41 × 10^–4^	0.016	Annual
M2W	1.41 × 10^–4^	0.016	Winter
M2S	1.41 × 10^–4^	0.016	Summer
K1M2	7.29 × 10^–5^ 1.41 × 10^–4^	0.031 0.016	Annual

## Results

### Observations

**Figure 2:**
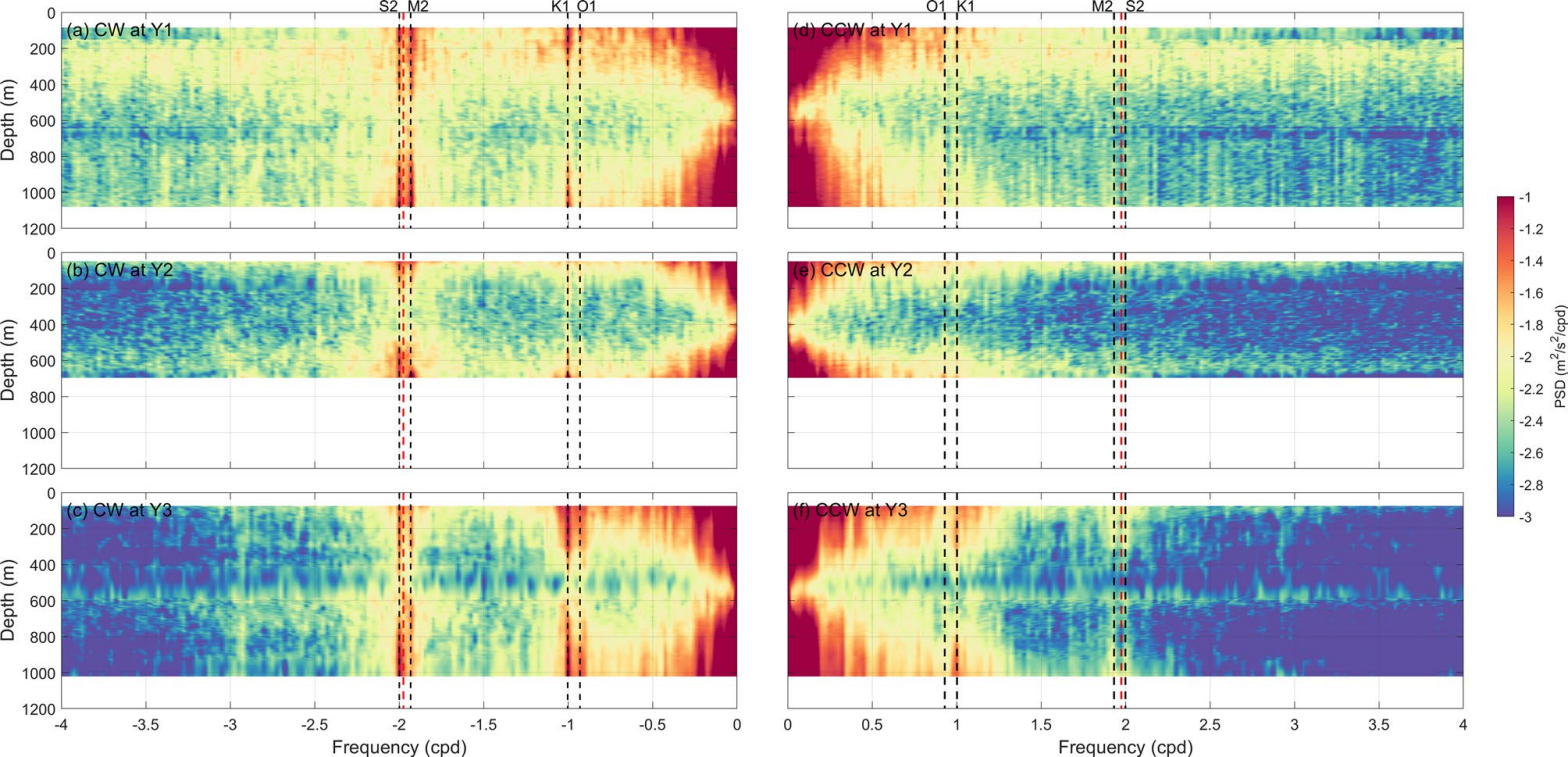
Rotary frequency spectra of horizontal baroclinic velocities at the three moorings (shading, unit: m^2^/s^2^/cpd). CW and CCW components are shown in (**a**–**c**) and (**d**–**f**), respectively. Tidal frequencies are indicated by black dashed lines, while local inertial frequency by red dashed lines.

Rotary frequency spectra are calculated with horizontal baroclinic currents at the three moorings. Here, the baroclinic current is directly calculated by removing vertical-averaged current from raw observations (see Text S1 in supplementary information). A 40-day window with half overlapping is adopted to calculate the spectra, yielding a resolution of 0.025 cpd, which is sufficient to separate the local inertial frequency with semidiurnal tidal frequencies. As shown in Fig. 2, the baroclinic motions at the three moorings are similar, which are dominated by low-frequency background currents. Spectral densities at diurnal and semidiurnal frequencies are obviously smaller than those at frequencies lower than 0.5 cpd. In addition, no significant peaks are found in the tri-diurnal and quarter-diurnal frequency bands at the three moorings, suggesting the lack of high-frequency motions. In terms of rotation, the clockwise (CW) components are dominant for both the diurnal and semidiurnal bands, of which large spectral densities are found near the surface and bottom.

**Figure 3:**
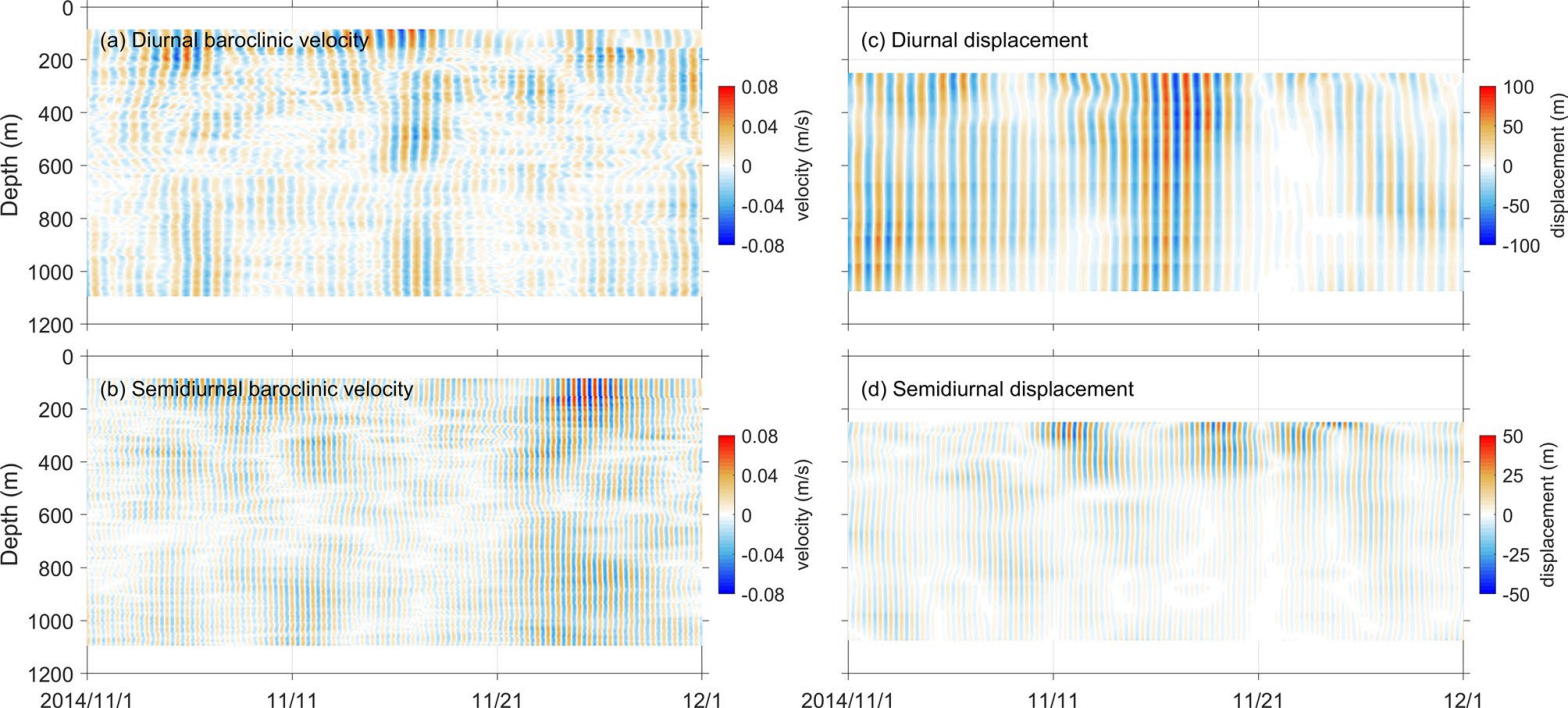
Diurnal and semidiurnal (**a** and **b**) baroclinic currents (shading, unit: m/s) and (**c** and **d**) isothermal displacements from 1 November to 31 December 2014 at Y1.

One-month segments of baroclinic currents and isotherm displacements corresponding to the diurnal and semidiurnal internal waves are shown in Fig. 3, from which their depth-time structures could be preliminarily illustrated. Results at Y1 are taken as an example. On the whole, the diurnal and semidiurnal baroclinic currents do not show visible difference in the magnitude, whereas the diurnal displacements are much greater than the semidiurnal ones. As shown in Fig. 3c, the diurnal displacement could reach 100 m, while the isotherm displacement corresponding to semidiurnal internal waves is much weaker, especially in the deep water (Fig. 3d). Similar phenomenon also exists at the other moorings (Figs. S5 and S6 in Supplementary information).

**Figure 4:**
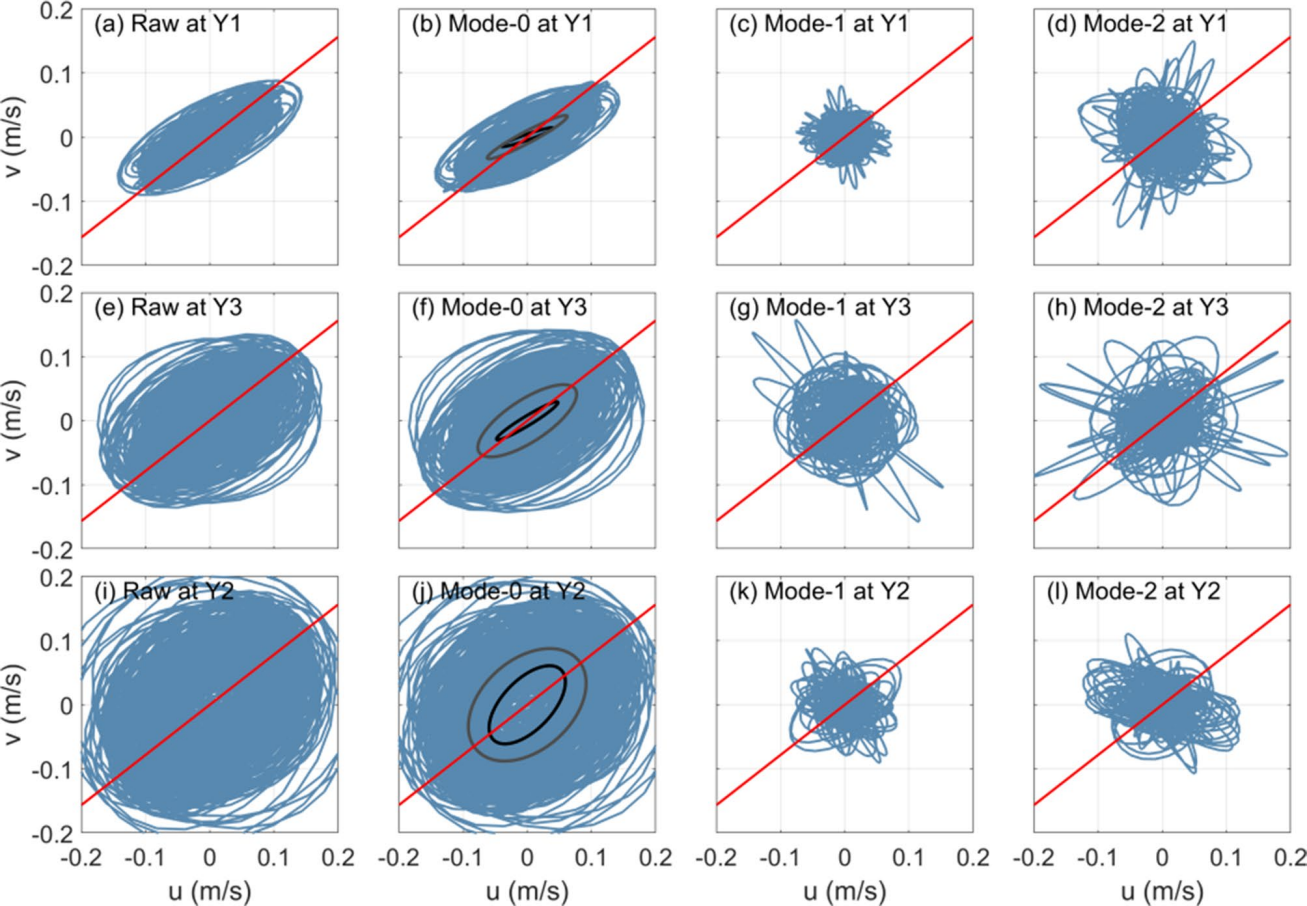
Hodographs of (**a**, **e** and **i**) depth-averaged raw diurnal currents and amplitudes of (**b**, **f** and **j**) mode-0, (**c**, **g** and **k**) mode-1 and (**d**, **h** and **l**) mode-2 diurnal currents at moorings Y1 (upper panel), Y3 (middle panel) and Y2 (lower panel). The red lines denote the cross-isobath directions as denoted in Fig. 1b. The black ellipses in (**b**, **f** and **j**) are the K_1_ tidal ellipses extracted from Arc5km2018.

**Figure 5:**
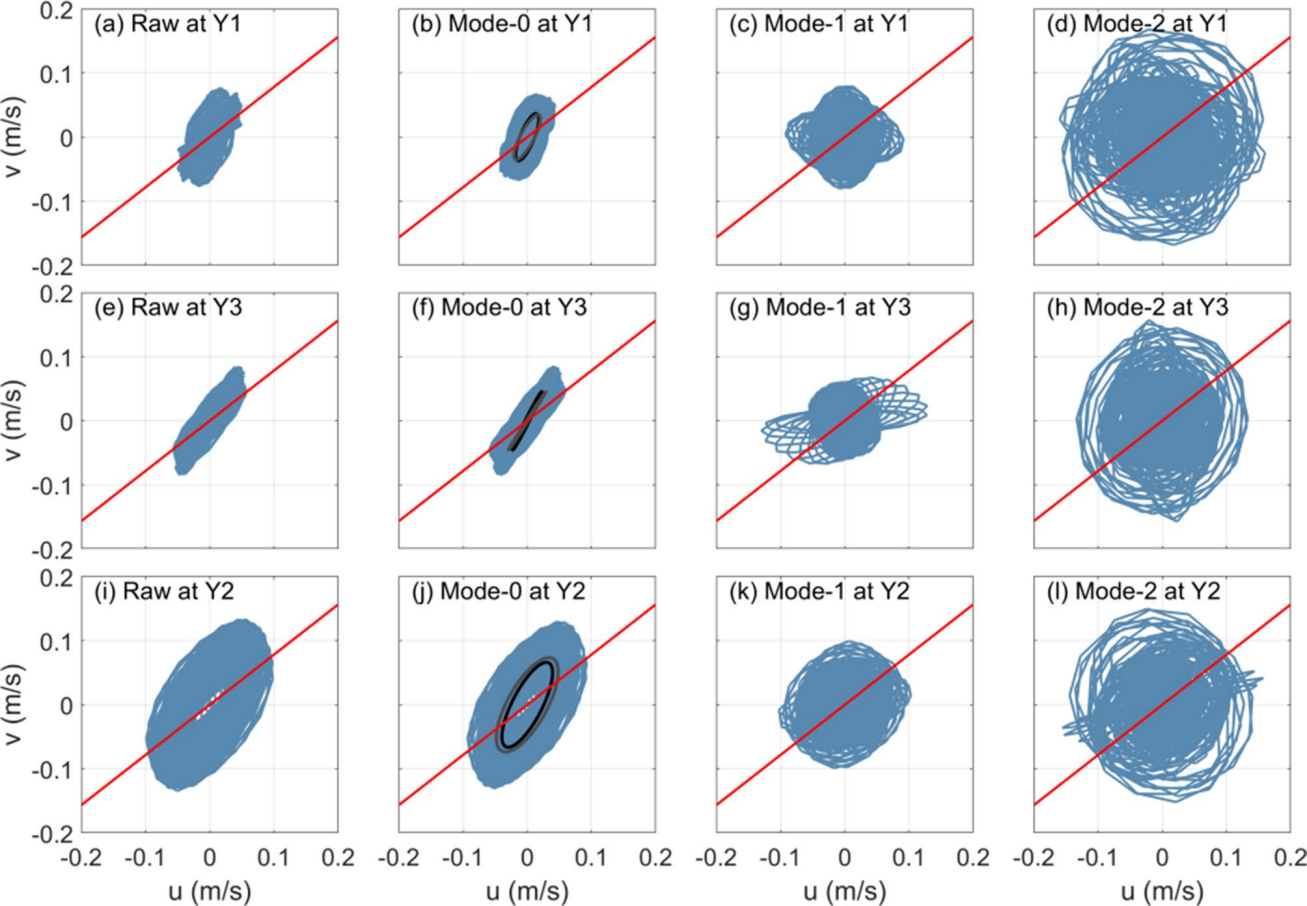
Same as (Fig. 3) but for semidiurnal currents. The black ellipses in (**b**, **f** and **j**) are the M_2_ tidal ellipses extracted from Arc5km2018.

Then we examine the wave characteristics by hodographs of horizontal currents at three moorings (Figs. 4 and 5). For both diurnal and semidiurnal currents at the three moorings, the hodographs of raw filtered currents almost share the same pattern as those of the mode-0 current amplitude. This result indicates that both the diurnal and semidiurnal currents at the three moorings are dominated by the barotropic component. The semidiurnal barotropic currents are found to be weaker than the diurnal ones. Except the diurnal barotropic currents at mooring Y2, which exhibit a circular feature (Fig. 4j), the diurnal and semidiurnal barotropic currents at these moorings show obvious polarized features: The major axes of diurnal barotropic currents at moorings Y1 and Y3 are nearly aligned in the cross-isobath direction (38° east by north, red line in Figs. 4 and 5), while the major axes of semidiurnal barotropic currents at the three moorings are along 60 ^o^ east by north. This is favorable for the generation of internal tidal waves over topography according to the theory^52,53^. Note that the observed polarization features of diurnal and semidiurnal barotropic currents generally agree with those of the K_1_ and M_2_ constituents extracted from the Arc5km2018 (black ellipses in Figs. 4 and 5), respectively. Compared with the barotropic currents, baroclinic currents show different features. Both the mode-1 and mode-2 currents are not polarized; instead, they exhibit a circular feature, indicating that the diurnal or semidiurnal internal waves do not have a deterministic propagation direction at the moorings^54^.

**Figure 6:**
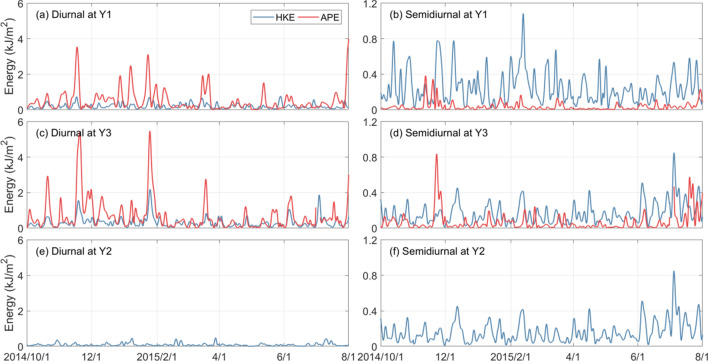
HKE (blue) and APE (red) of the diurnal (left panel) and semidiurnal (right panel) internal waves at moorings (**a** and **b**) Y1, (**c** and **d**) Y3 and (**e** and **f**) Y2. The HKE and APE have been lowpass filtered to eliminate the diurnal and semidiurnal oscillations of the diurnal and semidiurnal internal waves, respectively.

Figure 6 illustrates the HKE and APE of diurnal and semidiurnal internal waves at the three moorings, in which the first two baroclinic modes are considered. Note that we do not calculate the APE at mooring Y2 due to the bad modal decomposition result of vertical displacements there. On the whole, total energy (E = HKE + APE) of the diurnal internal waves are apparently greater than that of the semidiurnal ones in the deep region (moorings Y1 and Y3, Fig. 6 and Table 3). It is clearly shown that for diurnal internal waves, the APE is stronger than the HKE, whereas for semidiurnal internal waves, the HKE is dominant. As listed in Table 3, the annual mean HKE/APE ratios for diurnal internal waves at moorings Y1 and Y3 are 0.39 and 0.46. In contrast, the HKE/APE ratios for semidiurnal internal waves at moorings Y1 and Y3 are 6.78 and 2.42, which are generally comparable to those (2.55–6.70) estimated by Fer et al.^21^.

**Table 3:** Time-averaged diurnal and semidiurnal HKE, APE and HKE/APE ratio at the three moorings.

	Diurnal			Semidiurnal		
	HKE(J/m^2^)	APE(J/m^2^)	HKE/APE	HKE(J/m^2^)	APE(J/m^2^)	HKE/APE
Y1	196 ± 142	502 ± 598	0.39	244 ± 179	36 ± 49	6.78
Y3	309 ± 297	671 ± 870	0.46	162 ± 118	67 ± 105	2.42
Y2	94 ± 82	–	–	125 ± 109	–	–

Seasonal variations of diurnal and semidiurnal internal waves are different. For diurnal internal waves, their seasonal cycles could be preliminarily inferred, since the dominant APE is strong in winter but weak in summer (Figs. 6a and c). At mooring Y1 (Y3), the total diurnal energy is 0.90 (1.17) kJ/m^2^ in winter and 0.69 (1.07) kJ/m^2^ in summer. However, the situation is different for semidiurnal ones. Variations of semidiurnal internal waves are different at the three moorings, as the semidiurnal HKE in summer is a little enhanced at moorings Y3 and Y2 but somewhat weakened at Y1 (Figs. 6b,d and f). In addition, both the diurnal and semidiurnal energy do not exhibit apparent spring-neap cycles. By decomposing them into coherent and incoherent components^38,55–57^, it is found that their intermittence is caused by the incoherent components.

### Numerical simulations

**Figure 7:**
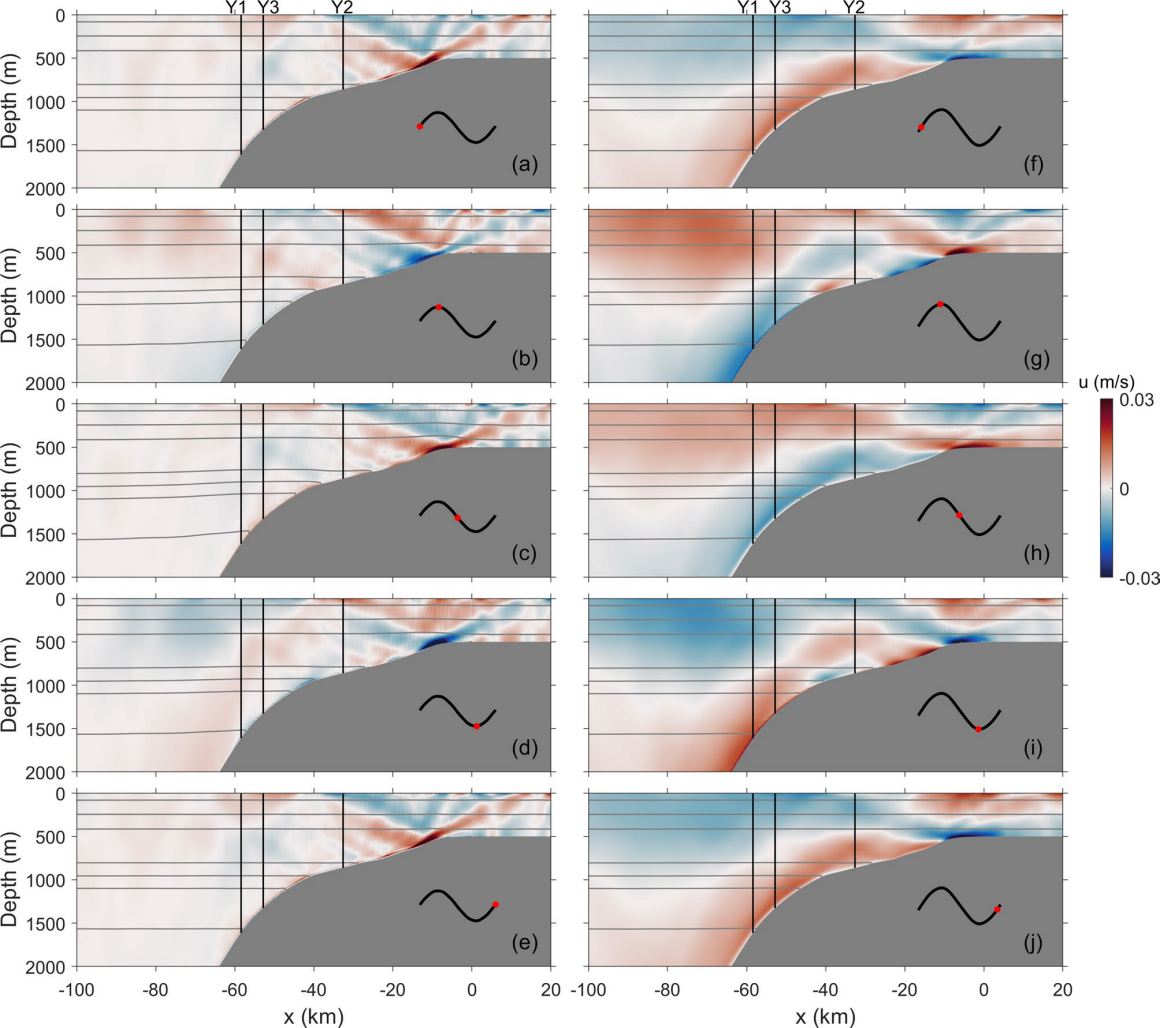
Snapshots for run (**a**–**e**) K1A and (**f**–**j**) M2A. Cross-isobath baroclinic currents are indicated by shadings (unit: m/s) and isotherms by gray contours. Phase for each snapshot is marked by red dot. Mooring locations are indicated by vertical black lines.

For further exploration on the internal wave dynamics on the southern slope of the YP, i.e., the different characteristics and HKE/APE ratios of diurnal and semidiurnal internal waves, numerical simulations are conducted. Figure 7 shows baroclinic currents and isotherm displacements of runs K1A and M2A. Here, baroclinic current is calculated by subtracting depth-averaged current from total velocity^58^. For run K1A, when onshore barotropic tidal flow accelerates, isotherms near the continental slope are lifted (Figs. 7a,b). Elevation of isotherms does not stop although the onshore tidal flow slackens (Figs. 7b,c). Instead, isotherms reach the highest position when onshore tidal flow decreases to zero (Fig. 7c). Thereafter, as the barotropic tidal flow turns, isotherms gradually move downward (Fig. 7d). They get back to the equilibrium positions when the tidal currents again slow down to zero (Fig. 7e). Different from those on the topographies at mid-latitude oceans, such oscillations of isotherms do not evolve into internal waves that are radiated offshore from the topography. Correspondingly, baroclinic currents in the offshore region (*x* < − 100 km) are rather weak.

Unlike the vertical displacements, notable values of baroclinic currents appear mainly around the shelf beak instead of the continental slope. Beam-like structures are observed at the shelf break (*x* = − 10 km), which are radiated both onshore and offshore (Fig. 7). Strength of the two beams is basically comparable. The offshore beam is nearly undetectable after one reflection at the sea surface. On the shallow shelf, due to the dissipation of high-mode waves, beam structure also becomes invisible^59,60^. Instead, low-mode waves going onshore are dominant (Figs. 7d,e).

When the M_2_ tidal forcing is imposed, the wave field exhibits different features. Upward and downward movements of isotherms exist, but their amplitudes are much smaller than those under the K_1_ tidal forcing (Fig. 7). In contrast, the baroclinic currents in run M2A are obviously greater than those in run K1A, and strong baroclinic currents can be found in the whole slope region. Two generation sites of internal wave beams are detected: one is at the base of continental slope (*x* = − 85 km) and the other is at the shelf break (*x* = − 10 km). In addition to the onshore-propagating waves on the shelf which are also found in run K1A, the offshore-propagating waves are clearly seen in this simulation. These results suggest that radiating waves could still be generated under sub-inertial forcing.

**Figure 8:**
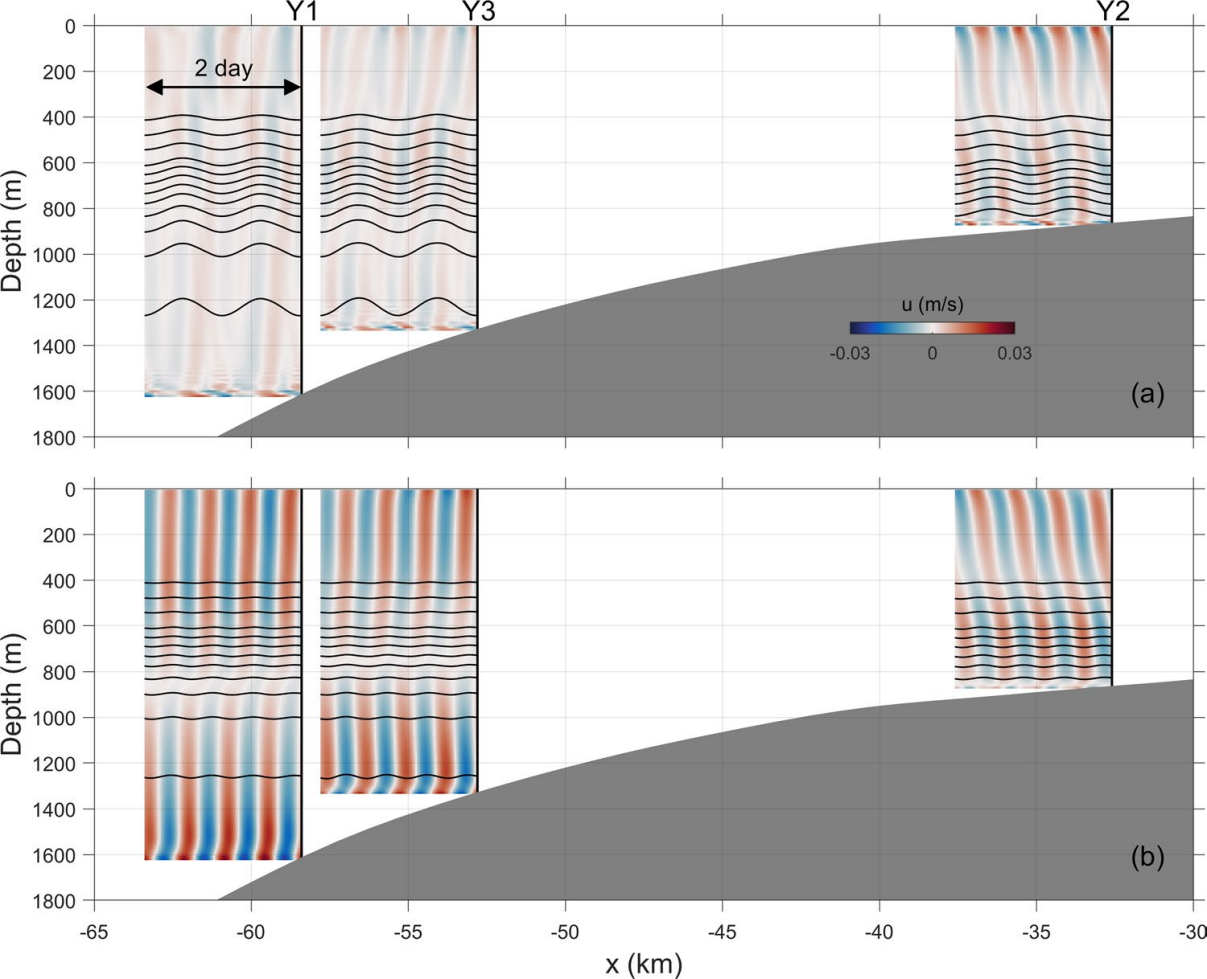
Time series of cross-shore baroclinic currents (shadings, unit: m/s) and isotherms (contours, unit: °C) at mooring locations for runs (**a**) K1A and (**b**) M2A.

Figure 8 presents the time-series of simulated baroclinic currents and isotherms at the three moorings. Under the K_1_ forcing, vertical displacements of isotherms show regular diurnal variability (Fig. 8a). Near the bottom, the amplitude of vertical displacements reaches nearly 100 m, and it gradually decreases with the distance from sea bottom. Oscillations of isotherms in shallow water are weaker than those in deep waters (e.g., at Y1 and Y3). These features are generally consistent with those captured by the moorings. As pointed out by Musgrave et al.^24^, this could be attributed to sub-inertial, bottom-trapped wave response. However, unlike the displacements, the baroclinic currents at all the moorings do not exhibit pronounced diurnal cycles. At Y1, the period of baroclinic currents cannot be directly determined from the time series. At Y3 and Y2, it is very interesting to find that semidiurnal variability dominates the baroclinic currents. Different from those in run K1A, both the displacements and baroclinic currents in run M2A exhibit remarkable semidiurnal cycles (Fig. 8b). It is easy to find that the amplitude of vertical displacements is much smaller than that in run K1A, which also agrees with the observational results. Nevertheless, the baroclinic currents in run M2A are stronger than those in run K1A, especially in the deep regions (Y1 and Y3), although the forcing amplitude in run M2A is smaller. Moreover, no visible high-frequency signals are detected in the baroclinic currents or vertical displacements in run M2A.

**Figure 9:**
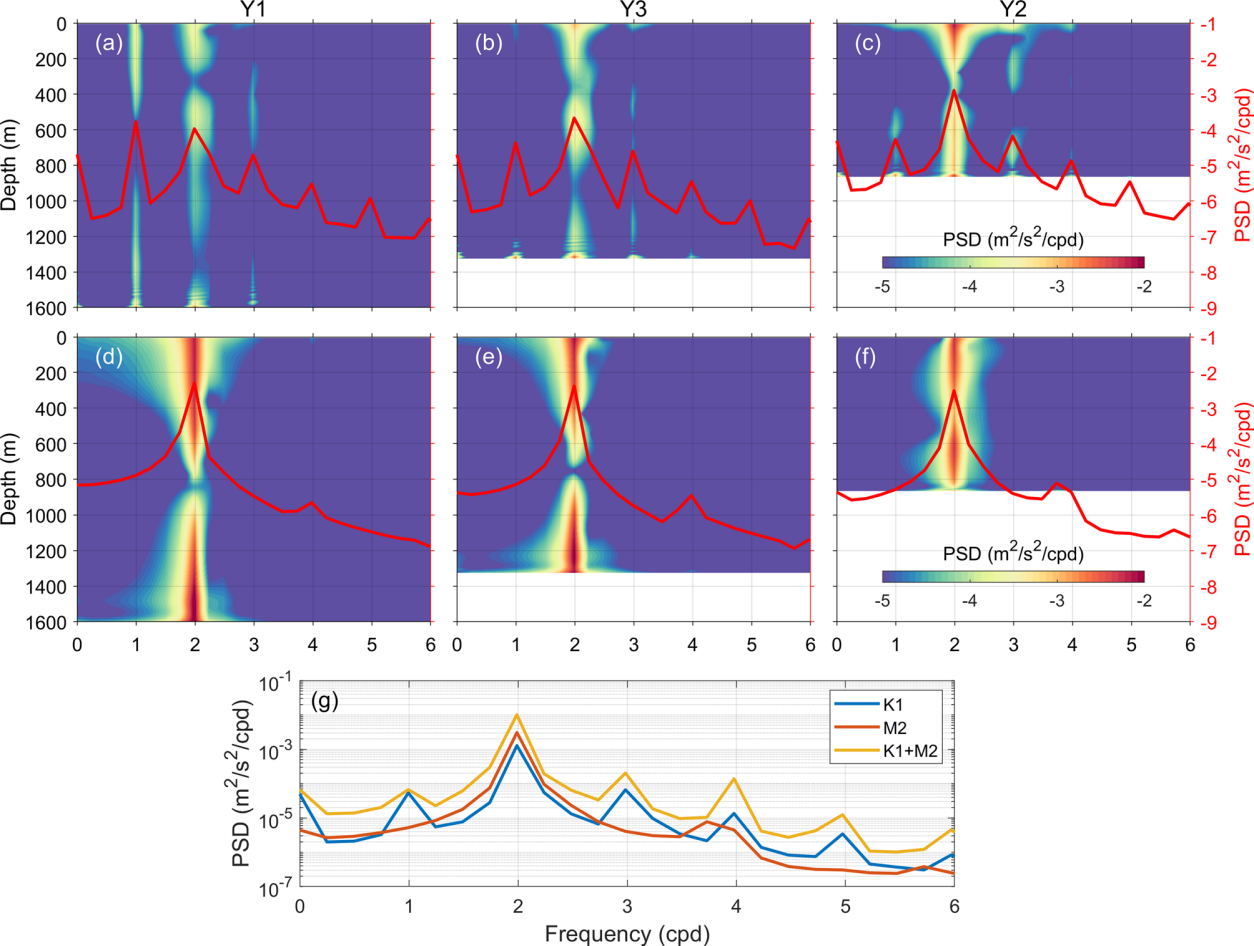
Power spectral densities (PSDs) of cross-isobath baroclinic currents at three mooring position (shading, unit: m^2^/s^2^/cpd) for (**a**–**c**) K1A (**d**–**f**) M2A. Depth-averaged PSDs are indicated by red curves in each subfigure. (**g**) Depth-averaged PSDs for different runs at Y2.

Since signals at frequencies beyond the forcing frequency are found in baroclinic currents in run K1A (Fig. 8a), the power spectral densities (PSDs) of cross-isobath baroclinic currents are calculated (Fig. 9). For run K1A (Figs. 9a–c), multi-frequency feature is found for the baroclinic currents at these moorings. At mooring Y1, the PSDs at the K_1_ frequency and its higher harmonics are generally comparable (Fig. 9a). At moorings Y3 and Y2, it is noteworthy that the largest PSD appears at the semidiurnal frequency, which is almost one order of magnitude greater than that at the diurnal frequency, i.e., the forcing frequency (Figs. 9b and c). Considering the locations of Y3 and Y2, such semidiurnal baroclinic motion could be attributed to the offshore beam generated at the shelf break (Fig. 7). In addition, peaks are also detected at other higher harmonics (3–5 cpd), of which the PSDs are generally in the same order of magnitude as that at the diurnal frequency. The mixture of signals at the K_1_ frequency and its higher harmonics make it difficult to determine the dominant period directly from the baroclinic currents (Fig. 8a). For run M2A, semidiurnal motions are dominant at all the moorings (Figs. 9d–f), in accordance with the results shown in Fig. 7b. Depth-averaged PSDs of higher harmonics are approximately two orders of magnitude lower than that at the semidiurnal frequency. Moreover, when both the K_1_ and M_2_ forcing are imposed (run K1M2), PSD at the semidiurnal frequency is higher than that with the single M_2_ forcing, suggesting the intensification of semidiurnal internal waves by the diurnal forcing (Fig. 9g).

The different features of high harmonics in runs K1A and M2A could be attributed to different tidal excursion $\varepsilon = \frac{{U_{0} }}{\omega L}$ for diurnal and semidiurnal tidal forcing, where *U*_0_ and *ω* are forcing amplitude and frequency, respectively, and *L* is the horizontal scale of topography. The total time derivation of tidal forcing *F*_T_ is expressed as^61,62^,

6 $$\frac{{dF_{T} }}{dt} = \frac{{\partial F_{T} }}{\partial t} + U\frac{{\partial F_{T} }}{\partial x},$$

For small *ε*, internal waves at the forcing frequencies are generated; but for large *ε*, the second advection term in Eq. (6) could be dominant, resulting in the generation of waves not only at the fundamental frequency but also at higher harmonics^24,37^. Because the K_1_ barotropic tidal current has a larger amplitude (Figs. 4 and 5) and lower frequency than the M_2_, the tidal excursion in run K1A is almost four times larger than that in run M2A. Hence, noticeable higher harmonics are detected in run K1A.

**Figure 10:**
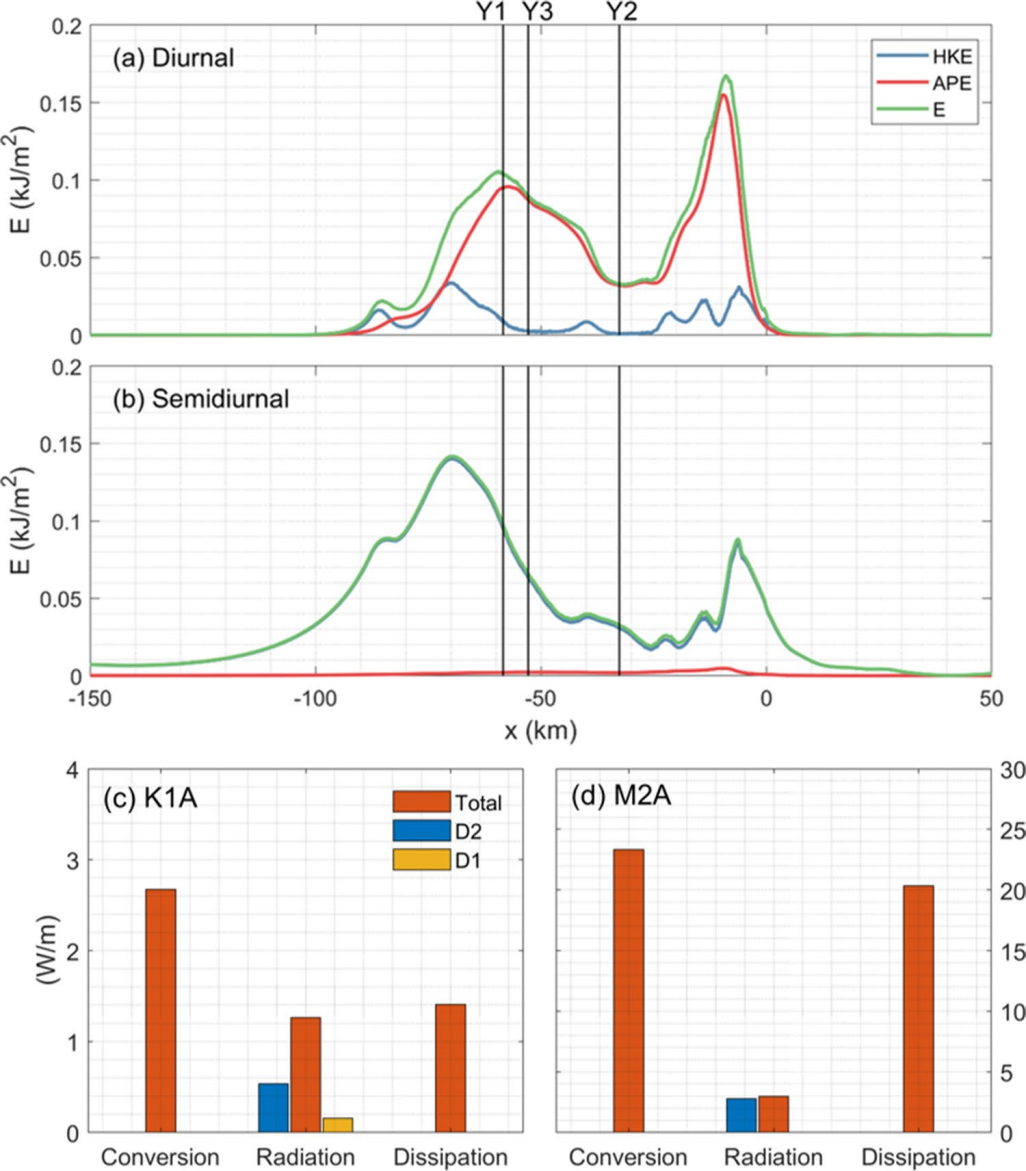
Depth-integrated (**a**) diurnal and (**b**) semidiurnal HKE, APE and E for runs K1A and M2A, respectively. Domain-integrated barotropic-to-baroclinic energy conversion, baroclinic energy radiation and dissipation for runs (**c**) K1A and (**d**) M2A. Red and green bars in (**c**) and (**d**) indicate filtered semidiurnal and diurnal energy radiation, respectively.

Then we calculate the internal wave energy from the model outputs. Because multi-frequency signals are found in the results of run K1A (Figs. 8 and 9), a bandpass filter is adopted to isolate the diurnal and semidiurnal signals. On the whole, the simulated results qualitatively agree with observations reported above: the APE is dominant for diurnal internal waves, whereas the HKE is dominant for semidiurnal internal waves (Fig. 10a and b). Both diurnal and semidiurnal energy decreases from Y1 to Y3, which is also consistent with observations (Table 3). There are two peaks of diurnal energy over the topography, with the higher one locating at the shelf break (Fig. 10a). Similar double-peak structure is also found for the semidiurnal energy; however, larger values of energy are detected on the slope region rather than around the shelf break.

Energy budget of internal waves is estimated following^63^:

7 $$\frac{\partial F}{{\partial x}} = C - D,$$

where the *F* is the baroclinic energy flux, *C* is the barotropic-to-baroclinic energy conversion rate, and *D* is the dissipation. The energy flux and conversion rate are calculated as

8 $$F = \int_{ - H}^{0} { < u^{\prime}p^{\prime} > dz},$$

and

9 $$C = < w_{bt} ( - H,t)p^{\prime}( - H,t) >,$$

where $\left\langle \cdot \right\rangle$ denotes an average over a tidal period, and pressure perturbation *p*’ and vertical barotropic velocity *w*_bt_ at the sea bottom are calculated as in previous studies^63–65^. Energy dissipation is calculated indirectly as the difference between energy conversion and flux divergence^21,24,66^. Note that the energy budget is integrated within -100 < *x* < 5 km, where the entire continental slope is covered. As shown in Figs. 10c and d, the semidiurnal tidal forcing yields much larger barotropic-to-baroclinic energy conversion, although its forcing amplitude is smaller than the diurnal one. According to Kerry et al.^63^, this could be explained by the phase difference between *w*_bt_(− *H*, *t*) and *p*’(− *H*, *t*), which almost approaches 90° in run K1A. A small fraction of energy is radiated away from the topography. In run K1A, nearly half of the radiated energy is attributed to semidiurnal waves, i.e., the higher harmonics, while the contribution of diurnal waves is actually limited (Fig. 10c). However, the situation is quite different for run M2A, where the fundamental (semidiurnal) waves play a dominant role in energy radiation (Fig. 10d). This result is consistent with the above spectral analysis results (Fig. 9).

A large proportion of energy is dissipated locally, especially for that in run M2A, which could explain the observed intense turbulent mixing on the YP^20,21,28^. Following previous studies^67^, we further examine the local dissipation coefficient *q* which is defined as the ratio of energy dissipation to conversion. Here, the values of *q* are 0.53 and 0.87 in runs K1A and M2A, respectively, which are much larger than those in mid-latitude oceans where strong tidal forcing exists, such as the Luzon strait (0.4^67^), the Hawaii Islands (0.19^68^) and the Mendocino Ridge (0.20–0.70^24,69^). This result highlights the role of diurnal and semidiurnal internal waves in driving mixing in the Arctic ocean.

## Discussions

### Discrepancies with linear theory

In the linear theory of internal waves, the governing equation in terms of stream function *ψ* is ^23,25,70^,

10 $$\frac{{\partial^{2} \psi }}{{\partial x^{2} }} - \frac{{\omega^{2} - f_{0}^{2} }}{{N^{2} (z) - \omega^{2} }}\frac{{\partial^{2} \psi }}{{\partial z^{2} }} = 0 ,$$

Because *ω* < *f*_0_ poleward of the critical latitude, the above equation is elliptic. As a result, corresponding solutions represent evanescent waves that are trapped near the topography. Nevetheless, the simulated results suggest the existence of freely propagating internal waves poleward of the critical latitude, of which the frequencies are lower than the local inertial frequency. Therefore, energy could be radiated away from the topography, although the proportion is lower than that in the mid-latitude oceans. Moreover, in the presence of strong tidal forcing, the radiating internal waves could further evolve into solitons^25,26,44^. This implies that the role of nonlinear terms should be taken into consideration.

Linear theory predicts that energy conversion poleward the critical latitude is proportional to $\sqrt {{{f_{0}^{2} } \mathord{\left/ {\vphantom {{f_{0}^{2} } {\omega^{2} }}} \right. \kern-\nulldelimiterspace} {\omega^{2} }} - 1}$^71^. In other words, low-frequency tidal forcing yields higher energy conversion for a certain latitude. But in our numerical results, it is found that diurnal energy conversion is much lower than the semidiurnal one, even though the diurnal forcing is stronger. This may suggest an overestimation of diurnal energy conversion poleward of the critical latitude^71^.

Moreover, both the observational and numerical results demonstrate a rather low HKE/APE ratio for diurnal internal waves, unsatisfying the theoretical relation^23^:

11 $$\frac{{{\text{HKE}}}}{{{\text{APE}}}} = \frac{{\omega^{2} + f_{0}^{2} }}{{\left| {\omega^{2} - f_{0}^{2} } \right|}} ,$$

In the mid-latitude oceans, the HKE/APE ratio deviating from the theoretical value may be related to standing waves^40,42,72,73^. However, as shown in the numerical simulations, the continental slope is the only generation site for diurnal internal waves (Fig. 7). There is no superposition of diurnal internal waves with different propagation directions and hence no standing waves. In other words, standing waves are not the cause for the low HKE/APE ratios of diurnal internal waves at the moorings.

The aforementioned discrepancies indicate that the linear theory of internal waves cannot be simply generalized to the Arctic Ocean. Modifications will be made in further theoretical work.

### Potential influence of other factors

**Figure 11:**
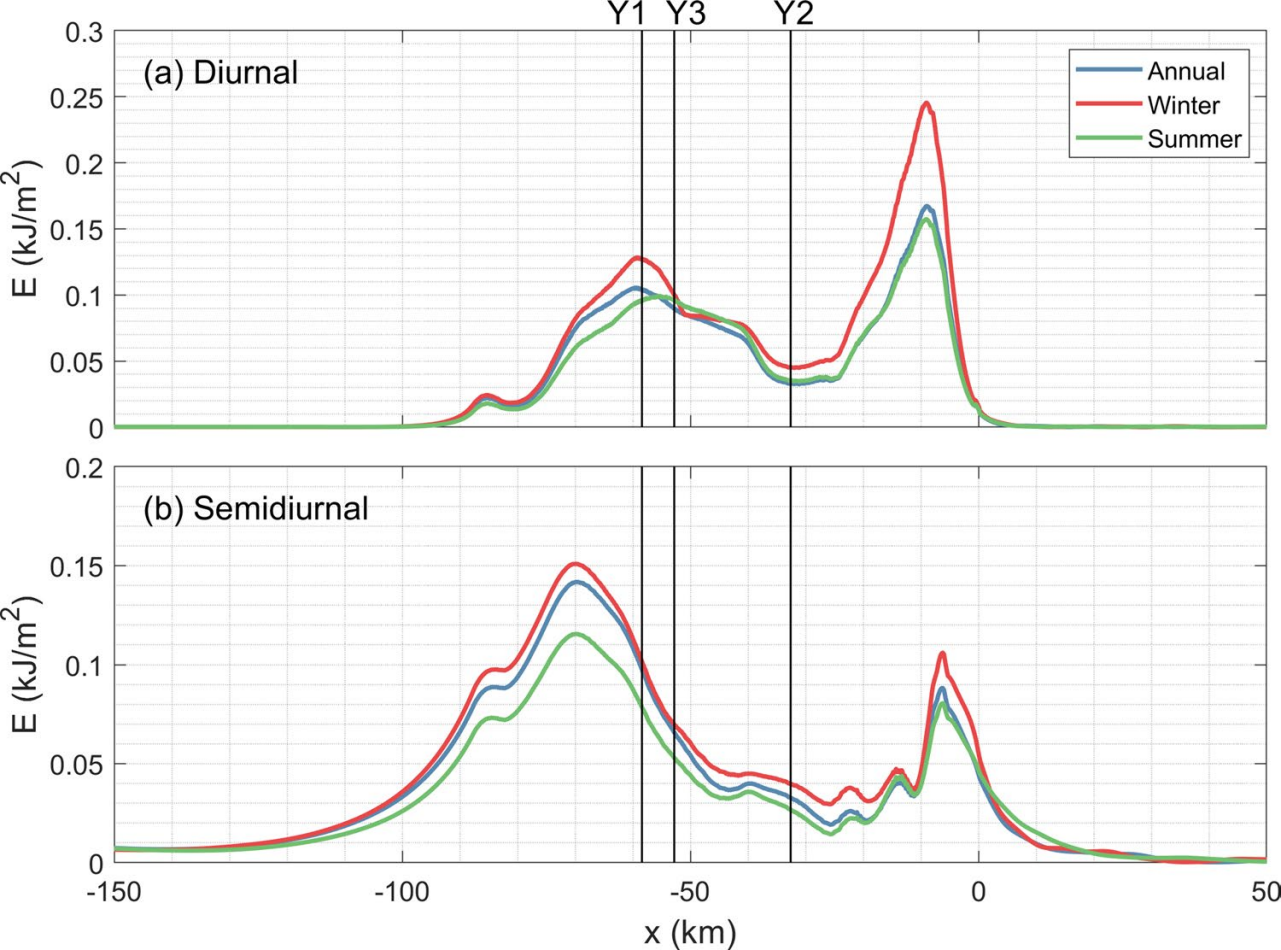
Depth-integrated (**a**) diurnal and (**d**) semidiurnal energy with different stratifications.

Annually averaged stratification is used in the simulations. Nevertheless, observations have indicated remarkable seasonal variability of stratification in this region^31^, which may affect the generated internal waves. To explore the impact of seasonal-varying stratifications, additional simulations with winter and summer stratifications are conducted (Table 1). As shown in Fig. 11, both diurnal and semidiurnal energy are strong in winter but weak in summer. For diurnal internal waves, the result is generally consistent with observations. This suggests that stratification could be a dominant factor controlling seasonal variability of diurnal internal waves. However, the simulated result disagrees with observations for semidiurnal waves, implying that their seasonal variation may be influenced by other factors. Because the semidiurnal frequencies are close to local Coriolis frequency, subtracting wind-generated near-inertial waves from semidiurnal band could be difficult. In other words, observed temporal variation of semidiurnal internal waves could be partially attributed to wind^35^, but quantitative contributions of wind and tide to semidiurnal internal waves still need further investigation.

In addition, the West Spitsbergen Current flows northward along the southern slope YP^28,31^ where the internal tidal waves are generated. According to the recent numerical work by He and Lamb^74^, such current considerably affects the generation of internal waves near the critical latitude. The background currents induce no-zero relative vorticity *ζ*, leading to variation of the effective Coriolis frequency, i.e., *f*_eff_ = *f*_0_ + *ζ*/2^39^. Therefore, they could potentially create blocking regions where radiating internal waves cannot exist, and hence influence tidal energy conversion^74^. Moreover, the two-dimensional simulations conducted in this study with horizontally homogeneous stratification are a large simplification for the real situation. Observational results have demonstrated that the hodographs of baroclinic currents are not polarized, suggesting that internal tidal waves could propagate not only cross-shore but also along-shore^21^. Along-shore propagation of internal waves can only be reproduced in a 3D model. For this circumstance, energy conversion and radiation would be different from those in 2D cases. Hence, three-dimensional numerical simulations with realistic tidal and subtidal forcings and stratification will be conducted for further investigation on internal wave dynamics at the YP in the future.

## Summary

In this study, based on long-term moored observations, the characteristics and variability of diurnal and semidiurnal internal waves on the southern slope of the YP are investigated. Observational results indicate that diurnal internal waves induce large isothermal displacements over 100 m, which are nearly one order of magnitude greater than those for semidiurnal internal waves. The total energy of diurnal internal waves is greater than that of semidiurnal ones. However, the diurnal internal waves have larger APE than HKE, whereas the semidiurnal internal waves have larger HKE than APE. This result is partly consistent with previous observations^21^ and partly divergent from the linear theory. Moreover, diurnal internal waves show seasonal variations, which are strong in winter but weak in summer. In contrast, the temporal variation of semidiurnal internal waves is somewhat complicated, which is bit different at the three moorings.

Further exploration on internal wave dynamics is carried out with a two-dimensional high-resolution numerical model. The simulated results qualitatively agree with observations: the APE (HKE) is dominant for diurnal (semidiurnal) internal waves. In addition, due to the large tidal excursion, pronounced higher harmonics (semidiurnal internal waves) are generated under the diurnal forcing, which contribute a large proportion to energy radiation. In contrast, when semidiurnal forcing is imposed, waves at fundamental frequency are dominant. By estimating energy budget over the topography, it is found that most of energy for both diurnal and semidiurnal internal waves is dissipated locally, yielding a large local dissipation coefficient over 0.5.

The internal waves in the AO exhibit different features from those at low latitudes. However, the dynamics lying behind has not been fully understood. We hope that the results shown in this study can deepen the understanding of internal waves in the AO to some extent. Further studies with comprehensive observations and three-dimensional high-resolution simulations will be carried out, so that we can have a better understanding on internal wave dynamics as well as their influences on heat budget and even climate in the AO.

## Supplementary Information

Supplementary Information.

## Data Availability

The moored observations are downloaded from http://metadata.nmdc.no/metadata-api/landingpage/ac35993ab477cb6b7d4a0d0070d57b43. The MULTIOBS data are downloaded from https://marine.copernicus.eu/. Bathymetry is obtained from https://ngdc.noaa.gov/mgg/global/. Source code of MITgcm is downloaded from http://mitgcm.org/ (version c66h).
