# Supplementary material for: Diurnal and semidiurnal internal waves on the southern slope of the Yermak Plateau
Source: Sci Rep. 2022 Jul 8;12:11682. doi: 10.1038/s41598-022-15662-0 (PMC9270340; doi:10.1038/s41598-022-15662-0)
Supplement: Supplementary file 1 — Supplementary Information. [file 41598_2022_15662_MOESM1_ESM.pdf]

Scientific Reports

Supplementary Information for

Diurnal and semidiurnal internal waves on the southern slope of the Yermak Plateau

Shuya Wang[1, 2+], Anzhou Cao[3+], Qun Li[2], Xu Chen[1]

[1] Key Laboratory of Physical Oceanography, Ocean University of China and Pilot National Laboratory for Marine Science and Technology, Qingdao, China

[2] MNR Key Laboratory for Polar Science, Polar Research Institute of China, Shanghai, China

[3] Ocean College, Zhejiang University, Zhoushan, China

Corresponding author: Qun Li (liqun@pric.org.cn)

[+] These authors contributed equally to this work.

Supplementary Information included in this file:

Supplementary Text S1

Supplementary Table S1

Supplementary Figures S1-S8

Text S1: Validation of modal decomposition results

In this study, modal decomposition was adopted to separate the barotropic and baroclinic components for the diurnal and semidiurnal motions. Therefore, it is necessary to validate the modal decomposition results before analyzing the diurnal and semidiurnal internal waves. Figure S7 shows the observed and reconstructed full-depth zonal currents (vertical displacements) based on modal decomposition as well as the reconstruction errors for the diurnal motion at mooring Y1 as an example. It should be noted that because the raw observations did not cover full water depth, the modal decomposition was divergent with the increase of N_m[1], and increasing the fitting weight at some depths[2] in Equation (1) did not solve this problem yet. Hence, N_m=2 was considered in the modal decomposition of this study to ensure the accuracy of results, the same as Alford and Zhao[3]. As shown in Figure S7, the reconstructed zonal currents and vertical displacements show good agreements with observations, respectively. At the same time, the reconstruction errors are generally one order of magnitude smaller than observations. These results suggest the reasonability and accuracy of the modal decomposition results at mooring Y1. Similar results can be found for the diurnal and semidiurnal motions at the other moorings except the vertical displacements at mooring Y2. Based on the modal decomposition results, the HKE, APE and baroclinic energy fluxes of the diurnal and semidiurnal motions at the moorings were calculated (Only the HKE was calculated at mooring Y2). Moreover, the vertical-averaged currents show good consistency with mode-0 currents calculated by modal decomposition (Figure S8), indicating the reasonability for results shown in Figure 2.

References:

1. Cao, A., B. Li, & X. Lv, Extraction of Internal Tidal Currents and Reconstruction of Full-Depth Tidal Currents from Mooring Observations. *J. Atmos. Ocean Tech.*, 32, 1414-1424 (2015).
2. Zhao, Z., M. H. Alford, J. A. MacKinnon, and R. Pinkel, 2010: Long-Range Propagation of the Semidiurnal Internal Tide from the Hawaiian Ridge. *J. Phys. Oceanogr.*, **40,** 713-736 (2010).

3. Alford, M. H., & Zhao, Z. Global Patterns of Low-Mode Internal-Wave Propagation. Part I: Energy and Energy Flux. *J. Phys. Oceanogr.*, **37**, 1829-1848 (2007).

Table S1. Information of the moorings and data

	**Y1**	**Y2**	**Y3**
Longitude	5°E 57.541’	5°E 48.733’	5°E 56.333’
Latitude	79°N 37.209’	80°N 03.876’	79°N 44.093’
Recovery depth (m)	1609	863	1327
Observation period	2014.09.10-2015.08.13	2014.09.10-2015.08.13	2014.09.11-2015.08.13
Temporal resolution (h)	1	1	1
Vertical resolution (m)	5	5	5

Figures

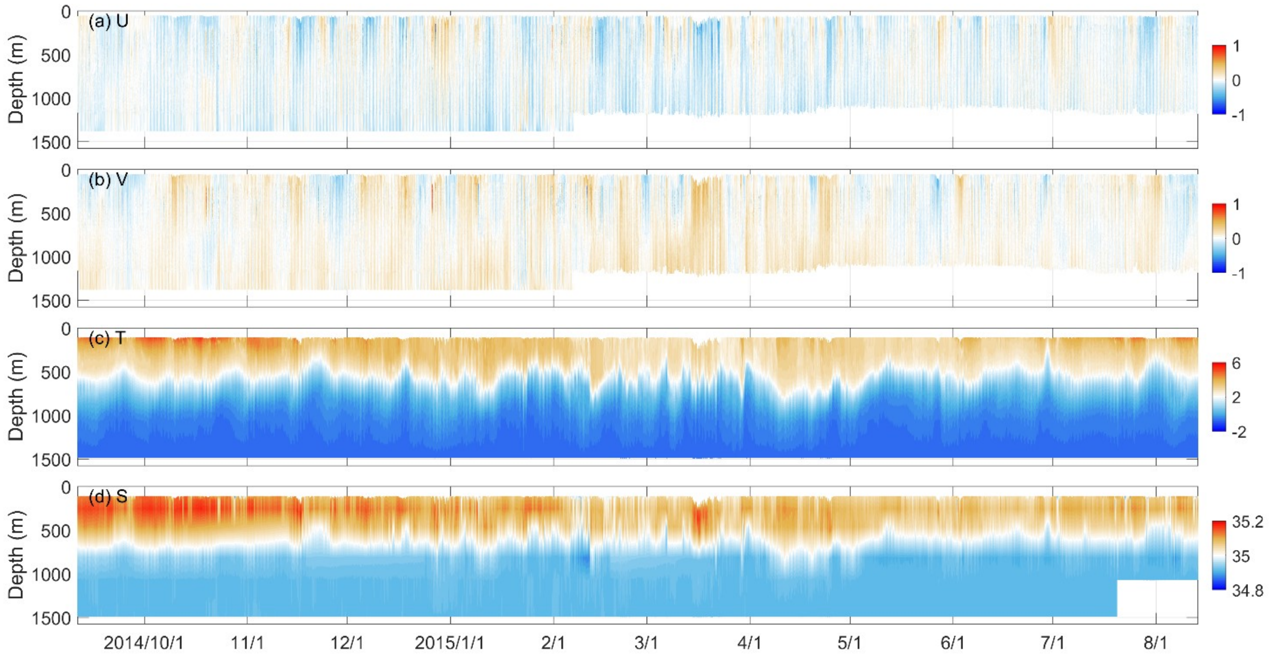

Figure S1. Raw observations of (a) zonal current (unit: m/s), (b) meridional current (unit: m/s), (c) temperature (unit: °C) and (d) salinity at mooring Y1.

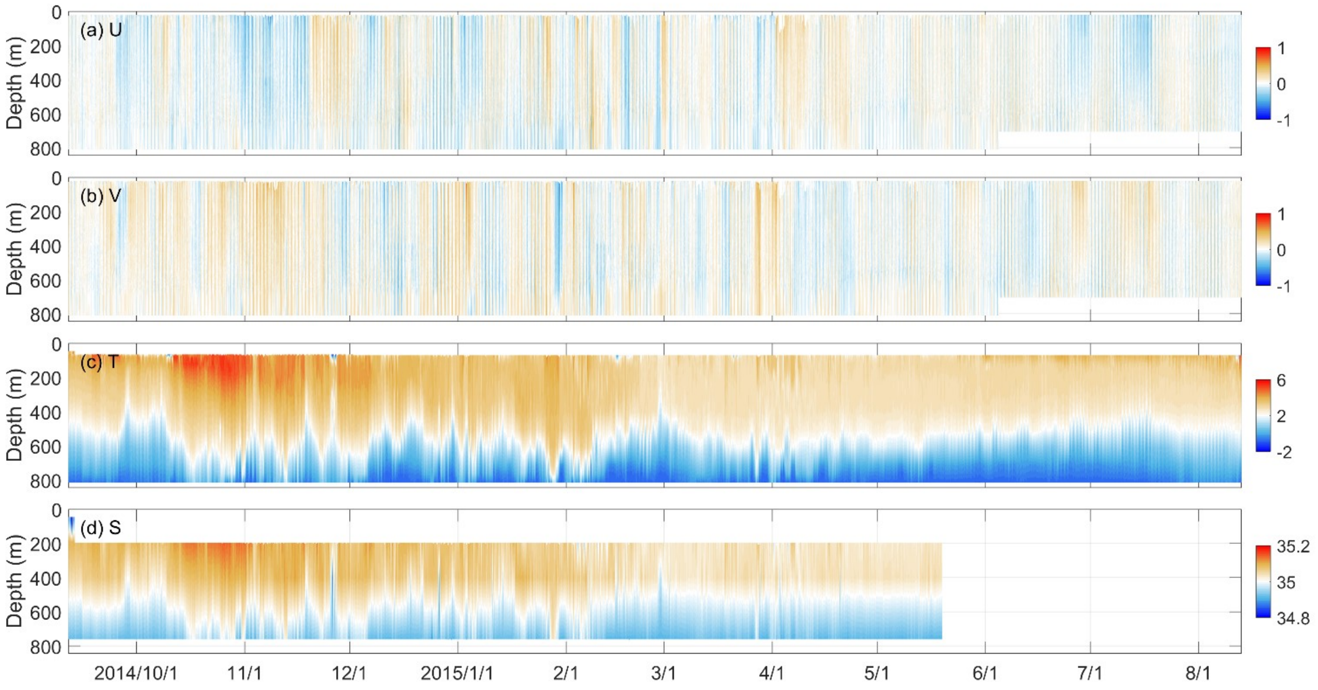

Figure S2. Same as Figure S1 but for Y2.

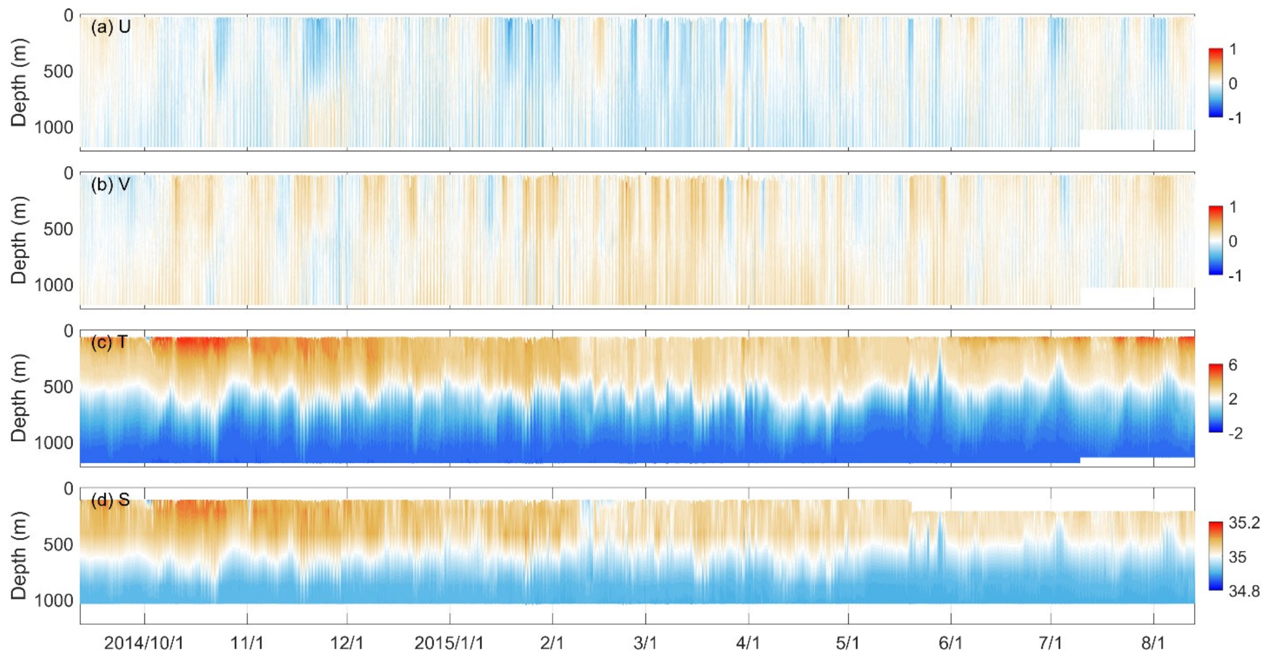

Figure S3. Same as Figure S1 but for Y3.

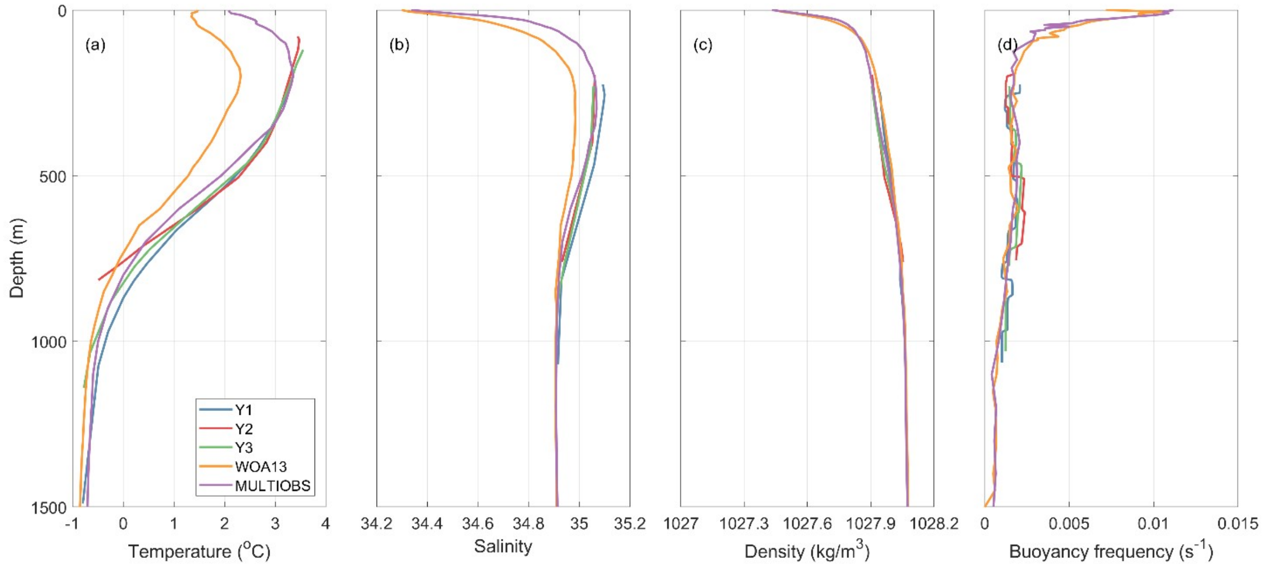

Figure S4. Comparison of moored observations, WOA13 and MULTIOBS for time-averaged (a) temperature, (b) salinity, (c) density and (d) buoyancy frequency.

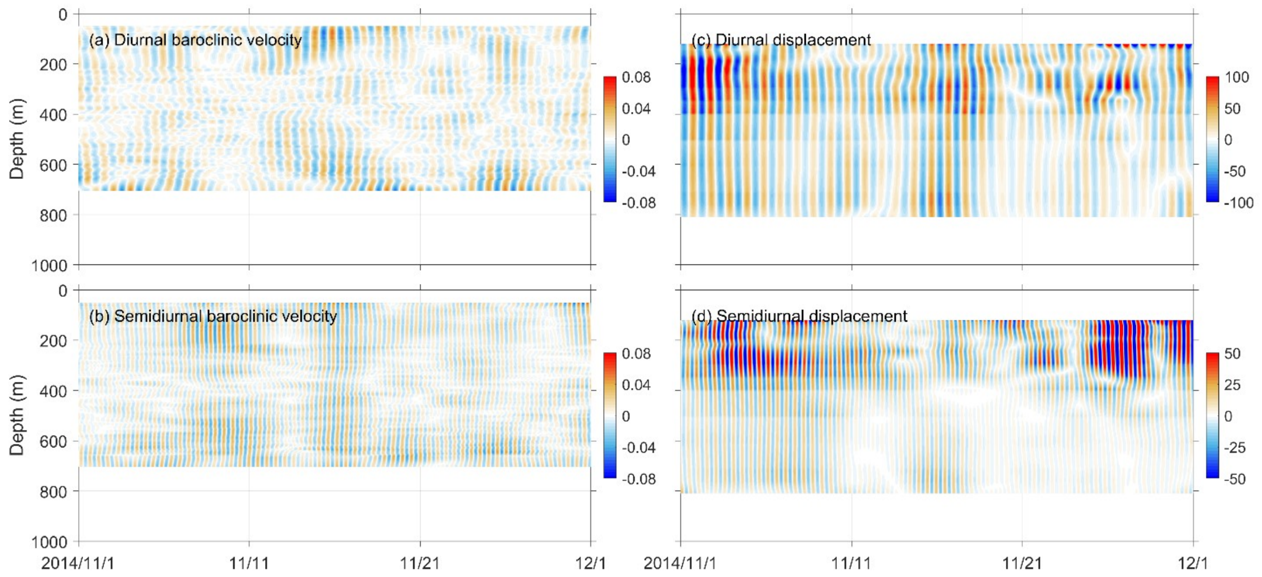

Figure S5. Diurnal and semidiurnal (a and b) baroclinic currents (shading, unit: m/s) and (c and d) isothermal displacements (shading, unit: m) from 1 November to 31 December 2014 at Y2.

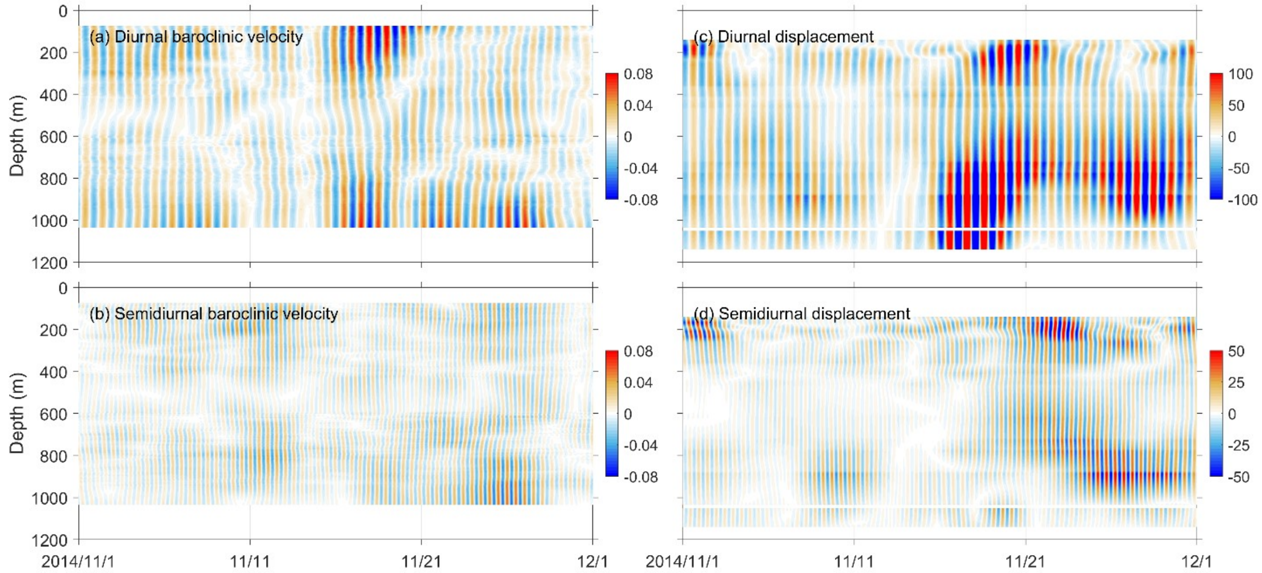

Figure S6. Same as Figure S5 but for Y3.

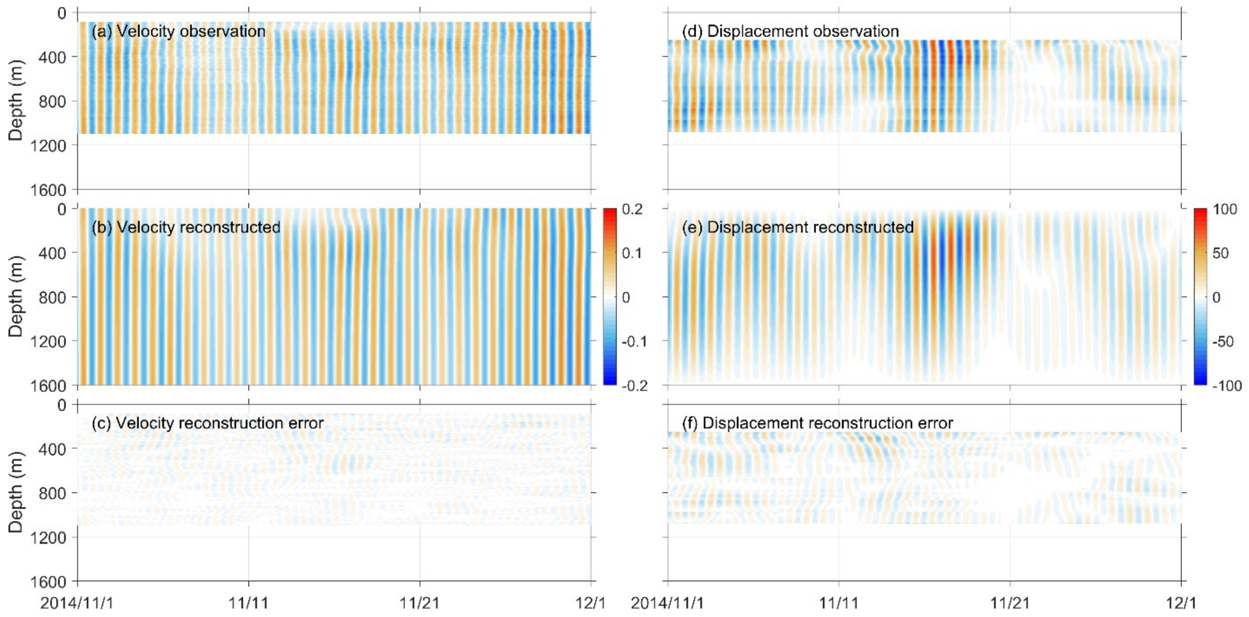

Figure S7. (a) Observed and (b) reconstructed diurnal currents (shading, unit: m/s) as well as (c) the reconstruction errors. (d-f) the same as (a-c) but for diurnal displacements (shading, unit: m).

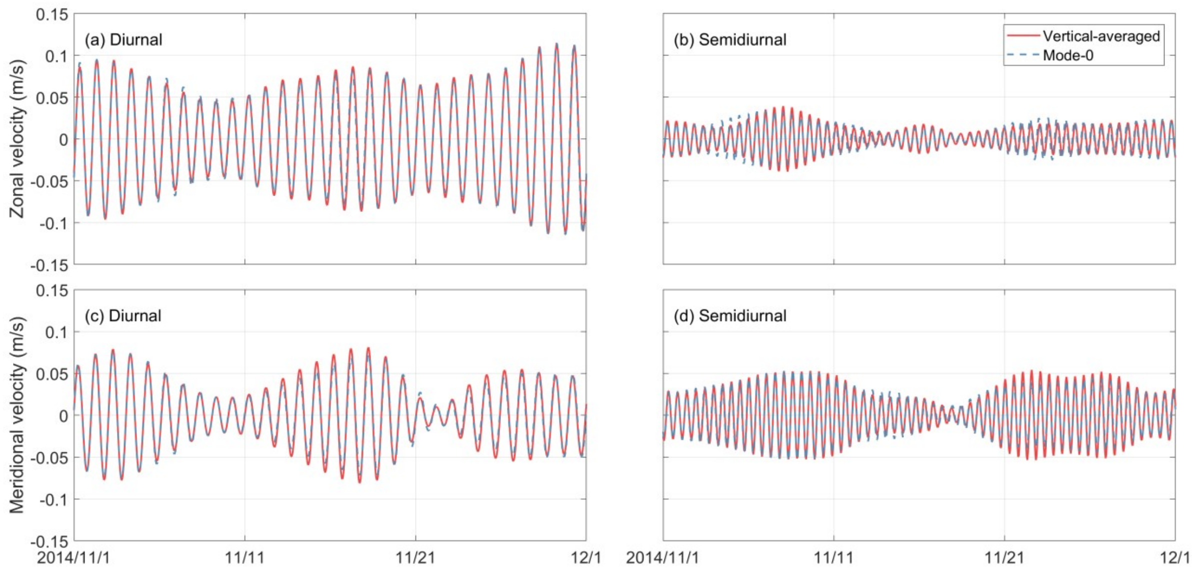

Figure S8. Comparison between vertical-averaged and mode-0 currents at Y1. (a-b) for zonal components, and (c-d) for meridional components.
